# Behçet syndrome: The disturbed balance between anti‐ (*CLEC12A*, *CLC*) and proinflammatory (*IFI27*) gene expressions

**DOI:** 10.1002/iid3.836

**Published:** 2023-04-12

**Authors:** Ali Kemal Oğuz, Çağdaş Şahap Oygür, Seda Taşır, Hilal Özdağ, Mehmet Nejat Akar

**Affiliations:** ^1^ Department of Internal Medicine, Division of General Internal Medicine Başkent University Faculty of Medicine Ankara Turkey; ^2^ Department of Internal Medicine, Division of Rheumatology Başkent University Faculty of Medicine Ankara Turkey; ^3^ Department of Biotechnology Ankara University Biotechnology Institute Ankara Turkey; ^4^ Department of Pediatrics TOBB University of Economics & Technology School of Medicine Ankara Turkey

**Keywords:** Behçet syndrome, *CLC*, *CLEC12A*, Galectin‐10, Gene expression, *IFI27*, Inflammation

## Abstract

**Introduction:**

Behçet syndrome (BS) is a chronic, multisystemic inflammatory condition with unanswered questions regarding its pathogenesis and rational therapeutics. A microarray‐based comparative transcriptomic analysis was performed to elucidate the molecular mechanisms of BS and identify any potential therapeutic targets.

**Methods:**

Twenty‐nine BS patients (B) and 15 age and sex‐matched control subjects (C) were recruited. Patients were grouped as mucocutaneous (M), ocular (O), and vascular (V) according to their clinical phenotypes. GeneChip Human Genome U133 Plus 2.0 arrays were used for expression profiling on peripheral blood samples of the patients and the control subjects. Following documentation of the differentially expressed gene (DEG) sets, the data were further evaluated with bioinformatics analysis, visualization, and enrichment tools. Validation of the microarray data was performed using quantitative reverse transcriptase polymerase chain reaction.

**Results:**

When *p* ≤ 0.05 and fold change ≥2.0 were chosen, the following numbers of DEGs were obtained; B versus C: 28, M versus C: 20, O versus C: 8, V versus C: 555, M versus O: 6, M versus V: 324, O versus V: 142. Venn diagram analysis indicated only two genes, *CLEC12A* and *IFI27*, in the intersection of M versus C ∩ O versus C ∩ V versus C. Another noteworthy gene appeared as *CLC* in the DEG sets. Cluster analyses successfully clustered distinct clinical phenotypes of BS. While innate immunity‐related processes were enriched in the M group, adaptive immunity‐specific processes were significantly enriched in the O and V groups.

**Conclusions:**

Distinct clinical phenotypes of BS patients displayed distinct expression profiles. In Turkish BS patients, expression differences regarding the genes *CLEC12A*, *IFI27*, and *CLC* seemed to be operative in the disease pathogenesis. Based on these findings, future research should consider the immunogenetic heterogeneity of BS clinical phenotypes. Two anti‐inflammatory genes, namely *CLEC12A* and *CLC*, may be valuable as therapeutic targets and may also help design an experimental model in BS.

## Graphical abstract

Behçet syndrome (BS), a chronic, multisystemic inflammatory condition seems to harbor an impaired balance between anti‐ and proinflammatory gene expressions. *CLEC12A*, *CLC*, and *IFI27* may be among these differentially expressed genes in Turkish BS patients. Another significant characteristic of BS is the observation of distinct expression profiles and molecular disease mechanisms in its clinical subgroups/phenotypes.

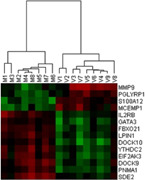

## 1 INTRODUCTION

Behçet syndrome (BS) is a chronic, multisystemic inflammatory condition characterized by a relapsing and remitting course.^1^ Initially defined by Dr. Hulusi Behçet as a “triple symptom complex” including oral and genital ulcers and uveitis, BS may present with diverse mucocutaneous, ocular, musculoskeletal, gastrointestinal, pulmonary, cardiovascular, and central nervous system manifestations, which are primarily vasculitic in origin.^2^, ^3^

Even though more than eight decades have passed since its definition, there are essential questions regarding BS that still need to be answered. The etiopathogenesis of the condition and its rational therapeutics are among these questions waiting for an answer. Three important properties of BS, namely (1) the divergent and sometimes paradoxical immunological findings observed in the studies, (2) the occurrence of diverse clinical features among different ethnic groups, and (3) the distinct clinical phenotype clusters within the syndrome, complicate the clarification of a sound, shared, and comprehensive etiopathogenesis for BS.^4^, ^5^, ^6^ Elucidating the disease mechanisms of BS at the molecular level may enable the scientific community to find the answers to the questions mentioned above, including the pathogenesis of BS, and may help develop novel, effective, and safer treatment approaches for the syndrome.

Previously, by borrowing the microarray data (Gene Expression Omnibus [GEO] data repository, GEO accession GSE17114) of the study by Xavier et al.,^7^ our group has demonstrated the presence of significant differences in gene expression and disease pathways between mucocutaneous, ocular, and vascular BS groups in a Portuguese BS patient population.^5^ Additionally, in the same study, four functional gene groups, namely (1) negative regulators of inflammation (*CD69*, *CLEC12A*, *TNFAIP3*), (2) neutrophil granule proteins (*LTF*, *OLFM4*, *AZU1*, *MMP8*, *DEFA4*, *CAMP*), (3) antigen processing and presentation proteins (*CTSS*, *ERAP1*), and (4) regulators of immune response (*LGALS2*, *BCL10*, *ITCH*, *CEACAM8*, *CD36*, *IL8*, *CCL4*, *EREG*, *NFKBIZ*, *CCR2*, *CD180*, *KLRC4*, *NFAT5*) were shown to be potentially instrumental in BS immunopathogenesis.^5^

In our present study, we performed a microarray‐based comparative genome‐wide expression analysis in Turkish BS patients and a sex and age‐matched healthy control group. We aimed (1) to elucidate the molecular disease mechanisms in Turkish BS patients, (2) to document any discrepancies between BS clinical phenotypes/patient subgroups regarding these disease mechanisms, and (3) to identify any potential therapeutic targets for BS.

## 2 PATIENTS AND METHODS

### 2.1 Approval of the ethics committee, centers participating in the study, and selection of patients with BS and control group individuals

The Ethics Committee of Ufuk University (Ankara, Turkey) approved the study with decision number 08065 dated 06.24.2009.

The BS patients included in this study consisted of BS cases visiting the outpatient clinics of Ufuk University Faculty of Medicine Department of Dermatology, Ankara University Faculty of Medicine Division of Rheumatology, and Ankara Numune Training and Research Hospital Rheumatology Clinic, for their routine follow‐up visits, who fulfilled the International Study Group for Behçet's Disease criteria for diagnosis of Behçet's disease and agreed to participate in the study by signing the relevant, informed consent form.^8^ BS patients with a current exacerbation of their disease other than a mucocutaneous involvement, a concomitant second inflammatory disorder, and any concurrent infectious or malignant diseases were excluded from the study.

The control group individuals included in the study were chosen to be compatible with the BS group regarding their age and gender. They were selected among individuals visiting Ufuk University Faculty of Medicine hospital checkup outpatient clinics, who were in absolutely good health, did not report any complaints consistent with an infectious or inflammatory disorder, did not have any current or past significant health issues, did not demonstrate any abnormal physical exam and laboratory findings, did not have a personal history of BS or a BS history in their families, and agreed to participate in the study by signing the relevant, informed consent form.

### 2.2 Collection of the blood samples and obtaining the clinical information of BS patients and control group individuals

Blood samples of BS patients and control group individuals were collected using PAXgene® Blood RNA Tube (PreAnalytiX®; Catalog no: 762165). A total of two tubes (samples) were obtained from each BS case and each control group individual included in the study, one for use during the study and the other as a precautionary measure. The blood samples collected were preserved at room temperature for the first 24 h, at −20°C for the next 24 h, and then at −80°C until the day they were to be analyzed, following the manufacturer's protocol (PAXgene® Blood RNA Tube Handbook).

The relevant clinical information of the BS cases and the control group individuals included in the study were obtained using the specifically designed “Case Report Form” (Supporting Information: 1 File). Because of major medical and ethical considerations, BS cases enrolled did not discontinue their current medications during the study.

### 2.3 Description of BS clinical phenotypes

The BS patients included in the study were grouped into four clinical phenotypes as mucocutaneous BS (M), ocular BS (O), vascular BS (V), and other BS (D) according to their individual clinical characteristics and the criteria presented in Table 1.^5^, ^9^, ^10^, ^11^, ^12^, ^13^

**Table 1:** Clinical characteristics defining individual Behçet syndrome clinical phenotypes.

Clinical phenotype	Defining clinical characteristics
Mucocutaneous BS	Only oral ulcers with genital ulcers and/or skin lesions and/or pathergy test positivity (i.e., isolated mucocutaneous findings) are present.
Ocular BS	In addition to mucocutaneous findings, there is only ocular BS involvement, which may present with different clinical manifestations.
Vascular BS	In addition to mucocutaneous findings, there is only vascular BS involvement without ocular involvement, which may present with different vascular lesions.
Other BS	In addition to mucocutaneous findings, there is ocular involvement and/or vascular involvement and/or musculoskeletal involvement and/or gastrointestinal system involvement and/or nervous system involvement.

Abbreviation: BS, Behçet syndrome.

### 2.4 Statistical analysis of key demographic characteristics

As the demographic data of the study did not follow a normal distribution, the median as a measure of central tendency together with the minimum and maximum values, and the Mann−Whitney *U* and the *χ* ^2^ tests to compare two independent groups were used. The *p* values of the analyses were presented with their absolute numerical values, and a *p* ≤ 0.05 was considered statistically significant. Statistical analyses of the demographic data were performed using “SPSS for Windows, Version 16.0” software (SPSS Inc. Released 2007. SPSS for Windows; Version 16.0.; SPSS Inc.).

### 2.5 RNA isolation and purification from peripheral whole blood samples

**Figure 1:**
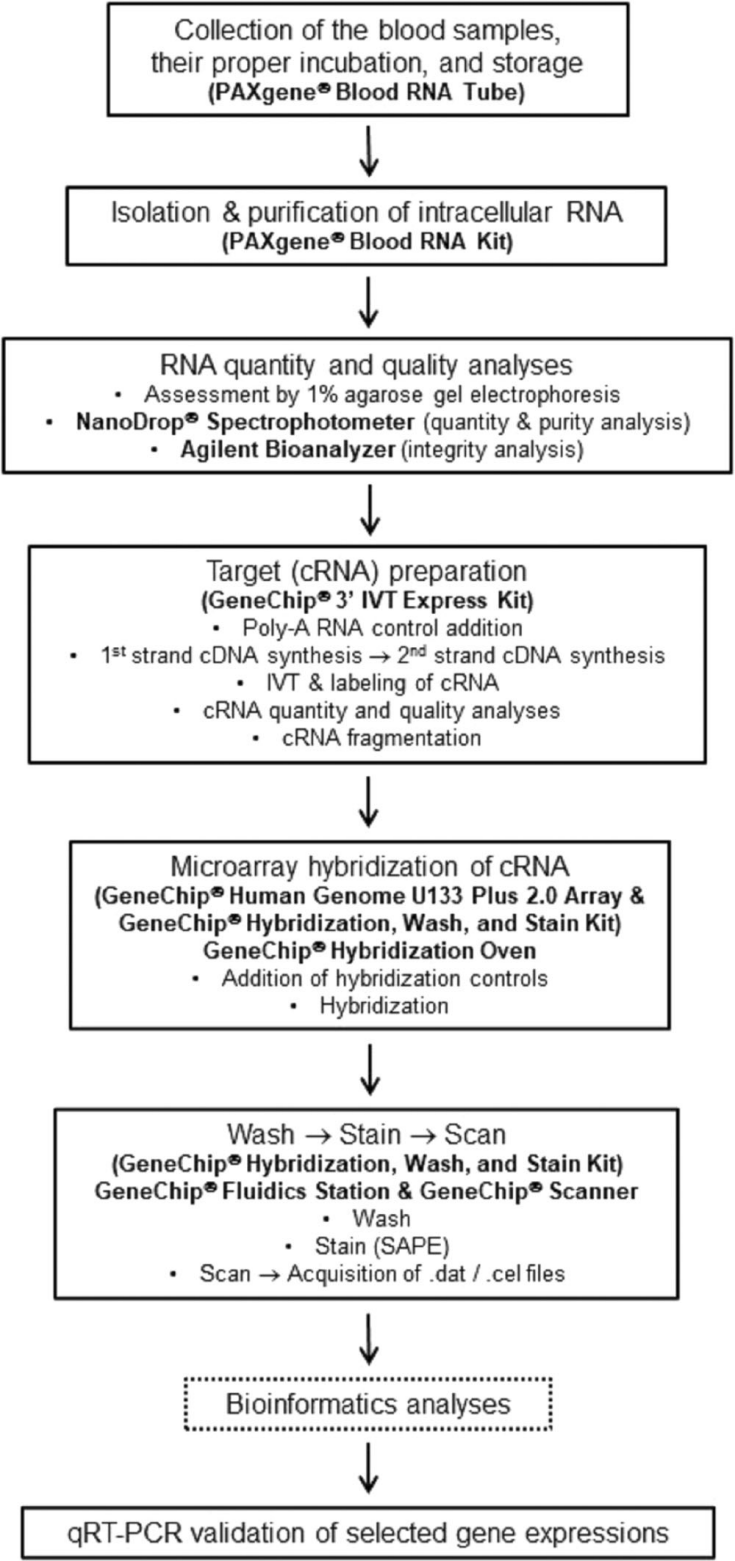
Flowchart of the in vitro experiments of the study. cDNA, complementary DNA; cRNA, complementary RNA; IVT, in vitro transcription; qRT‐PCR, quantitative reverse transcription polymerase chain reaction; SAPE, streptavidin, R‐phycoerythrin conjugate.

The flowchart summarizing the in vitro experiments of the study is presented in Figure 1.

RNA isolation & purification from blood samples collected for the study were performed using the PAXgene® Blood RNA Kit (PreAnalytiX®; Catalog no: 762174). Following the manufacturer's protocol, the tubes removed from the freezer just before the RNA isolation process were first equilibrated to room temperature and then kept at room temperature for an additional 2 h. All steps of the RNA isolation & purification process were performed in complete adherence to the protocol recommended by the manufacturer's, as described in the PAXgene® Blood RNA Kit Handbook. For every purified RNA sample, a 500 ng amount required for the downstream analysis was stored at −80°C, in addition to aliquots for RNA quantity/quality analyses and stocking purposes.

### 2.6 RNA quantity and quality analyses

The RNA samples isolated were run and evaluated in a 1% agarose gel. A260 and A280 values were measured using a nano‐spectrophotometer (NanoDrop® 2000/2000c Spectrophotometer; Thermo Fisher Scientific®). Consequently, they were analyzed by automated electrophoresis (2100 Bioanalyzer®; Agilent®) and their RNA integrity number (RIN) values were calculated. For the nano‐spectrophotometric and the automated electrophoretic evaluations, the protocols defined by the manufacturer's were followed strictly (Thermo Fisher Scientific® NanoDrop® 2000/2000c Spectrophotometer V1.0 User Manual; Agilent® RNA 6000 Nano® Kit Guide). The agarose gel images of randomly selected 20 RNA samples are shown in Supporting Information: 2 Figure. Concentration values, A260/A280 ratios, and RIN values of the purified RNA samples are presented in Supporting Information: 3 File.

### 2.7 Synthesis of amplified, biotin‐labeled, and fragmented target RNA

**Figure 2:**
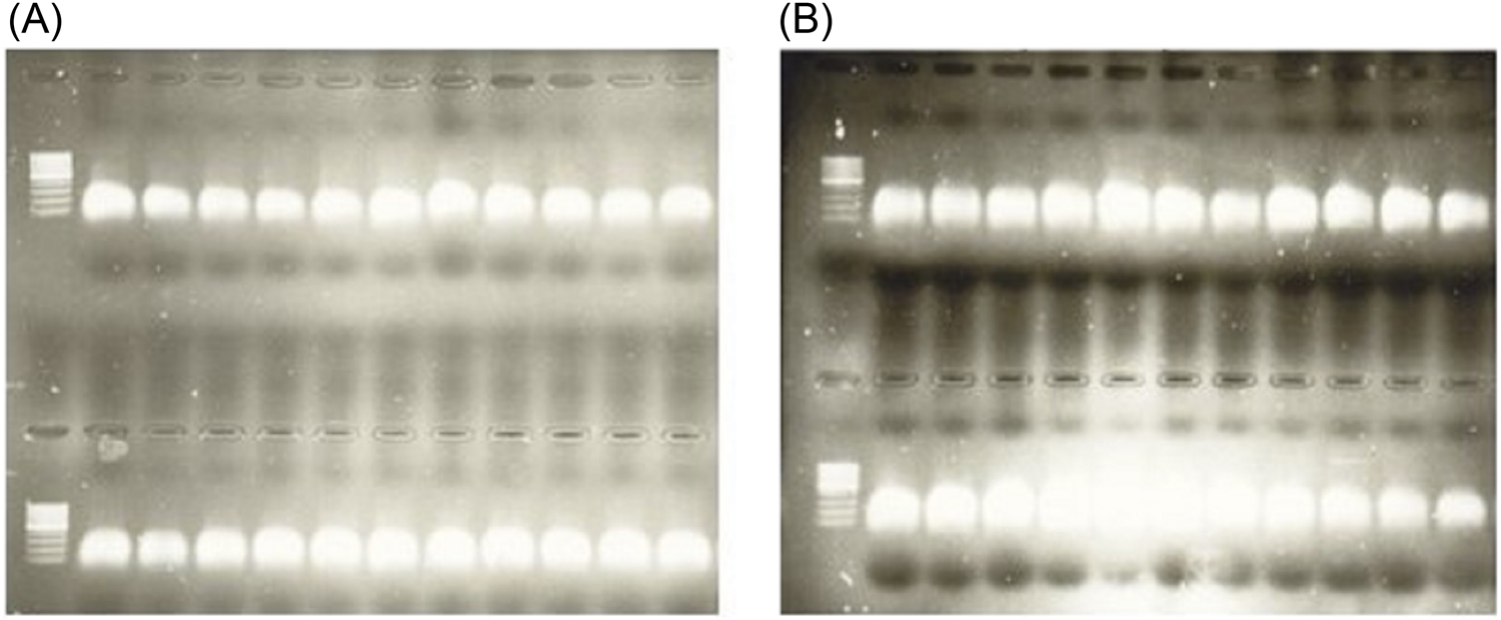
Gel images of the 44 target RNA samples following the fragmentation step. (A) Upper row, from left to right, “Ladder,” V1, V2, M1, O1, M2, D1, D2, V3, V4, O2, O3; bottom row, from left to right, “Ladder,” O4, V5, O5, D3, V6, V7, M3, V8, M4, M5, D4. (B) Upper row, from left to right, “Ladder,” M6, M7, M8, V9, D5, D6, D7, C3, C1, C2, C4; bottom row, from left to right, “Ladder,” C5, C6, C7, C8, C9, C10, C11, C12, C13, C14, C15. C1−C15, control individuals; D1−D7, other BS patients; M1−M8, mucocutaneous BS patients; O1−O5, ocular BS patients; V1−V9, vascular BS patients.

Before the microarray hybridization step, the target RNA was amplified, labeled with biotin, and then fragmented to increase the hybridization efficiency and obtain optimal results. Starting with an RNA sample of 500 ng, this step was carried out using the “GeneChip® 3' IVT Express Kit“ (Affymetrix®; Catalog no: 901229) and the protocol described by the manufacturer's (GeneChip® 3' IVT Express Kit User Manual). Biotin‐labeled amplified RNA was evaluated for quantity and purity by nano‐spectrophotometer (NanoDrop® 2000/2000c Spectrophotometer; Thermo Fisher Scientific®) and then fragmented and visualized on a 1% agarose gel. The concentrations and A260/A280 ratios of the amplified RNA samples are presented in Supporting Information: 3 File, and their gel images following the fragmentation step are shown in Figure 2.

### 2.8 Microarray hybridization

For microarray hybridization, “GeneChip® Human Genome U133 Plus 2.0” (Applied Biosystems®; Catalog no: 900466) microarrays were used, representing more than 47,000 transcripts/variants including 38,500 well‐defined genes of the human genome. Each microarray was loaded with a target RNA sample of 15 µg added to a hybridization cocktail prepared using the “GeneChip® Hybridization, Wash, and Stain Kit” (Applied Biosystems®; Catalog no: 900720). The preparation of the hybridization cocktail and the loading procedure of the microarrays were carried out in complete adherence to the manufacturer's protocol (GeneChip® Expression Analysis Technical Manual). Loaded microarrays were then allowed to hybridize in “GeneChip® Hybridization Oven” (Applied Biosystems®; Catalog no: 00‐0331) at a temperature of 45°C and 60 rpm rotation speed for 16 h.

### 2.9 Washing, staining, and scanning of microarrays

Microarrays, upon completion of the hybridization step, were subjected to washing and staining processes in the “GeneChip® Fluidics Station 450” (Applied Biosystems®; Catalog no: 00‐0079), using the relevant components in the “GeneChip® Hybridization, Wash, and Stain Kit” (Applied Biosystems®; Catalog no: 900720). Again, the protocol defined by the manufacturer's was precisely followed for this step (GeneChip® Expression Analysis Technical Manual).

Consequent to the washing & staining steps, the microarrays were transferred to and scanned on the “GeneChip® Scanner 3000” (Applied Biosystems®; Catalog no: 00‐0186) by using the “Affymetrix,® GeneChip® Command Console® 4.0” (AGCC) software. Upon completion of the scanning process, the AGCC software generated “.dat” and “.cel” files along with “.exp,” “.chp,” and “.rpt” files. Among these files, the.dat file contained the scanned microarray image, and the.cel file contained the light intensity (brightness) values of each probe set, defined by a grid. For the downstream bioinformatics analyses, these raw.cel files were used.

### 2.10 Quantitative reverse transcriptase polymerase chain reaction (qRT‐PCR) validation experiments for expression levels of selected genes

Validation experiments of this hybridization‐based expression study were performed using qRT‐PCR. For this purpose, the first 12 genes presented in the first column of Table 2 were selected, considering their fold change (FC) values, and potential significance to molecular disease mechanisms of BS. An effort was made to keep an absolute FC value of ≥3 for the genes to be selected. Based on the relevant literature, the peptidylprolyl isomerase B gene (*PPIB*) was chosen as the “housekeeping“ gene.^14^ Validation qRT‐PCR experiments were performed on a “LightCycler® 480” (Roche Applied Science®; Catalog no: 05 015 278 001) thermocycler, with 10 samples selected from each of the mucocutaneous BS, vascular BS, and healthy control groups. Each sample was run in triplicates. Details of the “RealTime Ready® Catalog Assays“ (Roche Applied Science®; Catalog no: 05 532 957 001) primers used during the experiments are also given in Table 2. “LightCycler® 480 Probes Master” (Roche Applied Science®; Catalog no: 04 707 494 001) was used to prepare the reaction medium, and the reaction setup was carried out following the protocol recommended by the manufacturer's. The “Delta‐Delta‐Ct” (ddCt) algorithm was used to calculate the qRT‐PCR‐based expression levels.^15^

**Table 2:** The 12 genes selected for the validation experiments and the details for their polymerase chain reaction primers.^a^

Gene	Primer	Sequence	Length	Tm	%GC	Position	Amplicon length
*IFI27*	Left	gtgttccacaggtcctctcc	20	59	60	420−439	75
	Right	ctcagtggtagatagaccctttcc	24	59	50	471−494	
*RSAD2*	Left	tctgcaatactatcccgttgg	21	59	48	1285−1305	63
	Right	ccacgtgcagcagaaaatc	19	60	53	1329−1347	
*IFI44L*	Left	tgctaaggagtatagcagatgaccta	26	59	42	4468−4493	77
	Right	ccacaacatcactctcactttaaga	25	59	40	4520−4544	
*CLEC12A*	Left	tttggggaaaaagcacctc	19	60	47	171−189	78
	Right	cagaaggcacagaagagtcaga	22	59	50	227−248	
*ARG1*	Left	caaggtggcagaagtcaaga	20	59	50	349−368	76
	Right	gcttccaattgccaaactgt	20	60	45	405−424	
*DEFA4*	Left	actccaggcaagaggtgatg	20	60	55	188−207	71
	Right	aaatagatatgtcctggtcttctgg	25	59	40	234−258	
*MS4A1*	Left	aaaaacagcatagcttatacatggac	26	59	35	1644−1669	65
	Right	gcctagagtgggagttaggaaaa	23	60	48	1686−1708	
*IGHM*	Left	acaggacttccttcccgact	20	60	55	90−109	69
	Right	gtgctgctgatgtcagagttg	21	59	52	138−158	
*TUBB2A*	Left	aaatatgtacctcgggccatc	21	60	48	235−255	66
	Right	tccagacctgacagagtcca	20	59	55	281−300	
*ORM1*	Left	tcgtgtacaccgattggaaa	20	59	45	596−615	101
	Right	ggctgtgtcctgctaggatt	20	59	55	677−696	
*IFIT1*	Left	tggcatgcacctgtagctt	19	60	53	2948−2966	64
	Right	gctccagcagtccactcac	19	59	63	2993−3011	
*CMPK2*	Left	agcatgttgtgtggcagaaa	20	60	45	1449−1468	67
	Right	aagagggtacgatggctgaa	20	59	50	1496−1515	
*PPIB*	Left	tctccgaacgcaacatga	18	59	50	230−247	109
	Right	ggccccttcttcttctcatc	20	60	55	319−338	

Abbreviations: %GC, GC‐content (guanine‐cytosine content); Tm, primer melting temperature (°C).

^a^ The “housekeeping” gene was chosen as peptidylprolyl isomerase B (*PPIB*).

**Figure 3:**
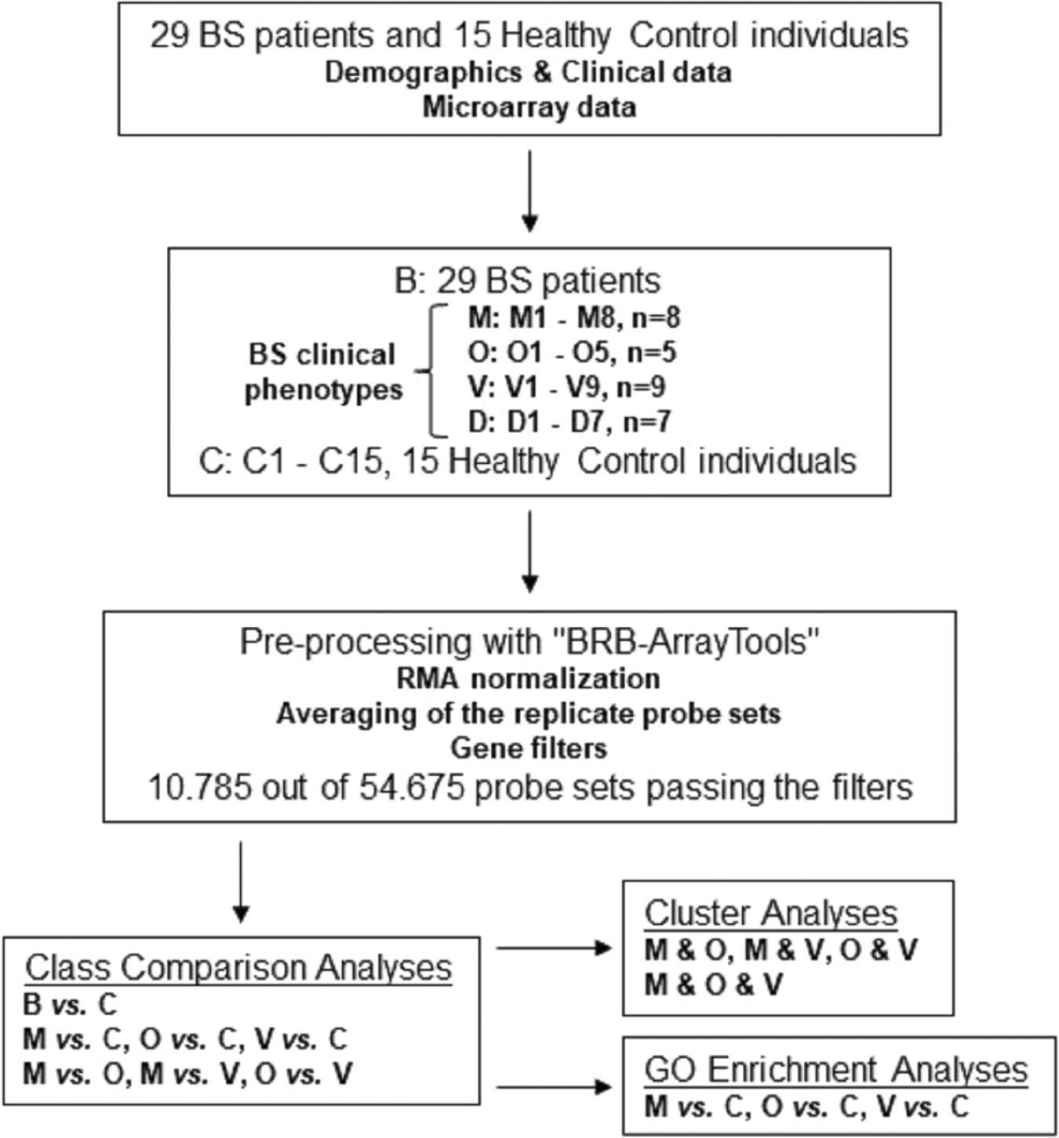
Flowchart summarizing the bioinformatics analyses of the study.B, the patient group including all of the BS patients; BS, Behçet syndrome; C, control group; D, Behçet syndrome group excluding the mucocutaneous, ocular, and vascular cases; GO, gene ontology; M, mucocutaneous Behçet syndrome group; O, ocular Behçet syndrome group; RMA, robust multiarray average normalization; V, vascular Behçet syndrome group.

### 2.11 Preprocessing of the microarray data

The metadata, raw, and final/normalized data of the study are deposited to the GEO repository (accession: GSE209567, date: 7.22.2022). The flowchart summarizing the bioinformatics analyses of the study is presented in Figure 3. The collation and all of the preprocessing steps of the microarray data were conducted using the “BRB‐ArrayTools v4.4.1 Stable release” developed by Dr. Richard Simon and the “BRB‐ArrayTools” development team (https://brb.nci.nih.gov/BRB-ArrayTools/). Initially, the raw microarray data existing as individual.cel files were collated using the data import function of the “BRB‐ArrayTools” software. Normalization of the imported microarray data was performed using the “Robust Multiarray Average” algorithm, which includes (1) background correction, (2) binary logarithmic transformation, and (3) quantile normalization.^16^ Following this normalization step, the replicate probe sets were averaged. Finally, gene filters were implemented, which helped to exclude genes that are not likely to be informative. For this purpose, a “minimum fold‐change filter” in addition to a “percent missing filter” were used, which were ready‐to‐use functions of the “BRB‐ArrayTools” software.

### 2.12 Bioinformatics analyses of the gene expression data

Class comparison analyses were performed using the “BRB‐ArrayTools v4.4.1” software (https://brb.nci.nih.gov/BRB-ArrayTools/). For the class comparison analyses, a two‐sample *t*‐test with a random variance model was implemented. The *p* value was chosen as ≤0.05 and the FC value as ≥2. For certain occasions, where a greater number of differentially expressed genes (DEG) are a prerequisite for optimal results (e.g., gene set enrichment analysis), an FC value ≥1.5 was also additionally used. The Venn diagram analysis of the findings of the class comparisons was performed with “Venny 2.1.0” developed by Juan Carlos Oliveros (https://bioinfogp.cnb.csic.es/tools/venny/).

For clustering analyses of BS cases, the built‐in clustering tools of “BRB‐ArrayTools” software and “Cluster 3.0” and the “TreeView” software packages were used (http://bonsai.hgc.jp/~mdehoon/software/cluster/software.htm#ctv).^17^ During the clustering analyses, both cases and genes were clustered together, and a hierarchical clustering algorithm using centered correlation metric and average linkage was implemented. The DEG sets used for clustering purposes were generated from the class comparisons M versus O, M versus V, and O versus V (two‐sample *t*‐test for all).

Gene ontology (GO) term enrichment analyses of the DEG sets were performed using the “WEB‐based Gene Set Analysis Toolkit” (WebGestalt) and specifically focused on the sub‐title of biological processes.^18^, ^19^ DEG sets obtained during M versus C, O versus C, and V versus C class comparisons were used for GO term enrichment analyses. The setup for the GO term enrichment analyses consisted of the hypergeometric test, the Benjamini−Hochberg correction for multiple comparison adjustment, and two as the minimum number of genes in a category.

## 3 RESULTS

### 3.1 Key demographics of the BS patients and the control subjects

The essential demographic characteristics of the BS patients and the control subjects are presented in Table 3. The groups B and C were similar with respect to their ages and genders.

**Table 3:** Key demographics of the Behçet syndrome patients and the control subjects.

	B (*n* = 29)	C (*n* = 15)	*p*	M^a^ (*n* = 8)	O^a^ (*n* = 5)	V^a^ (*n* = 9)	D^a^ (*n* = 7)
Age^b^ (min.−max./median)	18−58/32	19−57/36	0.711	25−57	18−58	27−56	18−40
Gender (male/female)	19/10	10/5	0.939	1/7	2/3	9/‐	7/‐

Abbreviations: B, the patient group including all of the Behçet syndrome patients; BS, Behçet syndrome; C, control group; D, Behçet syndrome group excluding the mucocutaneous, ocular, and vascular cases; M, mucocutaneous Behçet syndrome group; O, ocular Behçet syndrome group; V, vascular Behçet syndrome group.

^a^ For BS clinical phenotypes, only the minimum and the maximum ages are presented.

^b^ Age represents the age at the time of enrollment in the study.

### 3.2 Detailed demographic and clinical characteristics of the study group

The detailed demographic and clinical characteristics of the BS cases and the control subjects included in the study are presented in Table 4 (Supporting Information: 4 File). The group D (other BS) patients (D1−D7) were excluded from further analyses, due to their divergent clinical phenotypes, small sample sizes, and the study design.

**Table 4:** Demographic and clinical characteristics of the study group.

	Group	Gender	Age^a^	M	O	V	Additional involvement	BS clinical phenotype	EN
1	BS	Male	35	+	−	+	‐	Vascular	V1
2	BS	Male	43	+	−	+	‐	Vascular	V2
3	BS	Female	56	+	−	−	‐	Mucocutaneous	M1
4	BS	Male	24	+	+	−	‐	Ocular	O1
5	BS	Female	57	+	−	−	‐	Mucocutaneous	M2
6	BS	Male	32	+	−	+	MSS	Other	D1
7	BS	Male	18	+	−	−	GIS	Other	D2
8	BS	Male	28	+	−	+	‐	Vascular	V3
9	BS	Male	33	+	−	+	‐	Vascular	V4
10	BS	Female	58	+	+	−	‐	Ocular	O2
11	BS	Female	26	+	+	−	‐	Ocular	O3
12	BS	Female	47	+	+	−	‐	Ocular	O4
13	BS	Male	27	+	−	+	‐	Vascular	V5
14	BS	Male	18	+	+	−	‐	Ocular	O5
15	BS	Male	30	+	−	+	MSS	Other	D3
16	BS	Male	56	+	−	+	‐	Vascular	V6
17	BS	Male	28	+	−	+	‐	Vascular	V7
18	BS	Female	42	+	−	−	‐	Mucocutaneous	M3
19	BS	Male	32	+	−	+	‐	Vascular	V8
20	BS	Female	29	+	−	−	‐	Mucocutaneous	M4
21	BS	Female	46	+	−	−	‐	Mucocutaneous	M5
22	BS	Male	32	+	+	+	‐	Other	D4
23	BS	Female	25	+	−	−	‐	Mucocutaneous	M6
24	BS	Female	27	+	−	−	‐	Mucocutaneous	M7
25	BS	Male	53	+	−	−	‐	Mucocutaneous	M8
26	BS	Male	34	+	+	+	‐	Other	D5
27	BS	Male	22	+	‐	+	CNS	Other	D6
28	BS	Male	40	+	+	+	MSS	Other	D7
29	BS	Male	36	+	−	+	‐	Vascular	V9
30	Control	Male	19	−	−	−	‐	‐	C1
31	Control	Male	21	−	−	−	‐	‐	C2
32	Control	Male	27	−	−	−	‐	‐	C3
33	Control	Male	33	−	−	−	‐	‐	C4
34	Control	Male	34	−	−	−	‐	‐	C5
35	Control	Male	36	−	−	−	‐	‐	C6
36	Control	Male	37	−	−	−	‐	‐	C7
37	Control	Male	42	−	−	−	‐	‐	C8
38	Control	Male	48	−	−	−	‐	‐	C9
39	Control	Male	57	−	−	−	‐	‐	C10
40	Control	Female	24	−	−	−	‐	‐	C11
41	Control	Female	27	−	−	−	‐	‐	C12
42	Control	Female	42	−	−	−	‐	‐	C13
43	Control	Female	45	−	−	−	‐	‐	C14
44	Control	Female	57	−	−	−	‐	‐	C15

Abbreviations: BS, Behçet syndrome; CNS, central nervous system; EN, experiment name; GIS, gastrointestinal system; M, mucocutaneous involvement; MSS, musculoskeletal system; O, ocular involvement; V, vascular involvement.

^a^ Age represents the age at the time of enrollment in the study.

### 3.3 Number of probe sets used during comparative expression analyses

Following the preprocessing steps performed on the collated 44.cel files, 10,785 probe sets out of the 54,675 present on the “GeneChip® Human Genome U133 Plus 2.0” microarrays, passed the adjusted filters and were used during the subsequent bioinformatics analyses.

### 3.4 Findings of the class comparison analyses

Details regarding the number of the DEGs obtained during the class comparison analyses are shown in Table 5 (Supporting Information: 5 File).

**Table 5:** The number of the differentially expressed genes obtained during the class comparison analyses.

Classes compared	Number of differentially expressed genes					
	*p* ≤ 0.05, FC ≥ 1.5			*p* ≤ 0.05, FC ≥ 2.0		
	Total	Increased^a^	Decreased^b^	Total	Increased^a^	Decreased^b^
B versus C	662	150	512	28	13	15
M versus C	78	33	45	20	8	12
O versus C	118	24	94	8	4	4
V versus C	3475	529	2946	555	107	448
B versus C	662	150	512	28	13	15
M versus O	65	31	34	6	3	3
M versus V	2146	1708	438	324	222	102
O versus V	1125	998	127	142	114	28

Abbreviations: B, the patient group including all of the Behçet syndrome patients; C, control group; FC, fold change; M, mucocutaneous Behçet syndrome group; O, ocular Behçet syndrome group; V, vascular Behçet syndrome group.

^a^ Increased expression in the first class (e.g., B) compared to the second class (e.g., C).

^b^ Decreased expression in the first class (e.g., B) compared to the second class (e.g., C).

The top 20 (10 increased and 10 decreased) most differentially expressed genes, obtained by the class comparison analyses of the BS patient subgroups with the control group, are listed according to their FC values and presented in Table 6. Complete lists of the DEG sets can be found in the Supporting Information: 5 File.

**Table 6:** The top 20 most differentially expressed genes obtained during the class comparison analyses.^a^

Increased^b^				Decreased^c^			
**Gene symbol**		**FC**	***p***	**Gene symbol**		**FC**	***p***
M versus C							
1	*XIST* d	20.93	0.01129	1	*RPS4Y1* d	−11.82	0.00747
2	*IFI27* e	3.49	0.01443	2	*EIF1AY* d	−5.37	0.00837
3	*TMEM158*	2.59	0.01180	3	*DDX3Y* d	−5.21	0.00697
4	*PIGC*	1.88	0.04074	4	*TXLNGY* d	−3.64	0.00459
5	*EPSTI1*	1.85	0.03853	5	*KDM5D* d	−3.35	0.00966
6	*FRMD3*	1.83	0.00517	6	*CLEC12A* e	−3.17	0.00068
7	*IFIT3*	1.83	0.03644	7	*UTY* d	−2.39	0.02231
8	*OASL*	1.79	0.02884	8	*NAPSB*	−2.08	0.00083
9	*TRBV27*	1.75	0.04365	9	*LRRN3*	−2.02	0.02903
10	*CD274*	1.73	0.03022	10	*H3F3A*	−1.79	0.00004
O versus C							
1	*FKBP5*	2.68	0.00120	1	*CLEC12A* e	−2.78	0.01763
2	*IFI27* e	2.14	0.00319	2	*CLC*	−2.57	0.01603
3	*FECH*	2.14	0.03691	3	*CDKN1C*	−2.05	0.00849
4	*HEMGN*	2.02	0.03288	4	*KLRC3*	−1.98	0.03747
5	*ECHDC3*	1.88	0.04113	5	*COL18A1*	−1.94	0.00523
6	*RAP1GAP*	1.80	0.03904	6	*PRSS33*	−1.91	0.04309
7	*ZCCHC2*	1.78	0.00517	7	*C1orf21*	−1.86	0.01042
8	*IL13RA1*	1.76	0.00691	8	*LINC00926*	−1.84	0.00306
9	*MALAT1*	1.76	0.00925	9	*KLRF1*	−1.80	0.03415
10	*RNF182*	1.73	0.03784	10	*MRPL22*	−1.80	0.01458
V versus C							
1	*ARG1*	6.55	3.2e−06	1	*XIST* d	−6.29	0.04891
2	*DEFA4*	5.29	0.00057	2	*MS4A1*	−6.14	2.7e−05
3	*IFI27* e	4.34	0.00041	3	*IGHM*	−5.17	8.8e−05
4	*ECHDC3*	3.64	9.3e−05	4	*FCER1A*	−4.63	0.00051
5	*CA1*	3.58	0.00162	5	*LRRN3*	−4.38	4.0e−05
6	*IL1R2*	3.54	0.00028	6	*HLA‐DQA1*	−4.05	0.01032
7	*ANKRD22*	3.53	0.00137	7	*CLC*	−3.57	0.00011
8	*DAAM2*	3.50	0.00126	8	*PAX5*	−3.49	2.4e−05
9	*EIF1AY* d	3.50	0.01910	9	*RCAN3*	−3.45	1.4e−06
10	*ELANE*	3.48	0.00164	10	*BANK1*	−3.39	1.4e−05
					*CLEC12A*	−2.35	0.00696

Abbreviations: C, control group; FC, fold change; M, mucocutaneous Behçet syndrome group; O, ocular Behçet syndrome group; V, vascular Behçet syndrome group.

^a^ *p* ≤ 0.05 and FC ≥1.5 for all class comparisons (M versus C, O versus C, and V versus C).

^b^ Increased expression in the first class (e.g., M) compared to the second class (e.g., C).

^c^ Decreased expression in the first class (e.g., M) compared to the second class (e.g., C).

^d^ Sex chromosome (X or Y chromosome) gene appearing/interfering as a result of male/female ratio discrepancy between the compared classes.

^e^ The *IFI27* and *CLEC12A* genes which were the only two common genes in the DEG sets of the class comparisons M versus C, O versus C, and V versus C, were also among the top three most differentially expressed genes in these comparisons, specifically when the interfering sex chromosome genes were excluded from the list.

**Figure 4:**
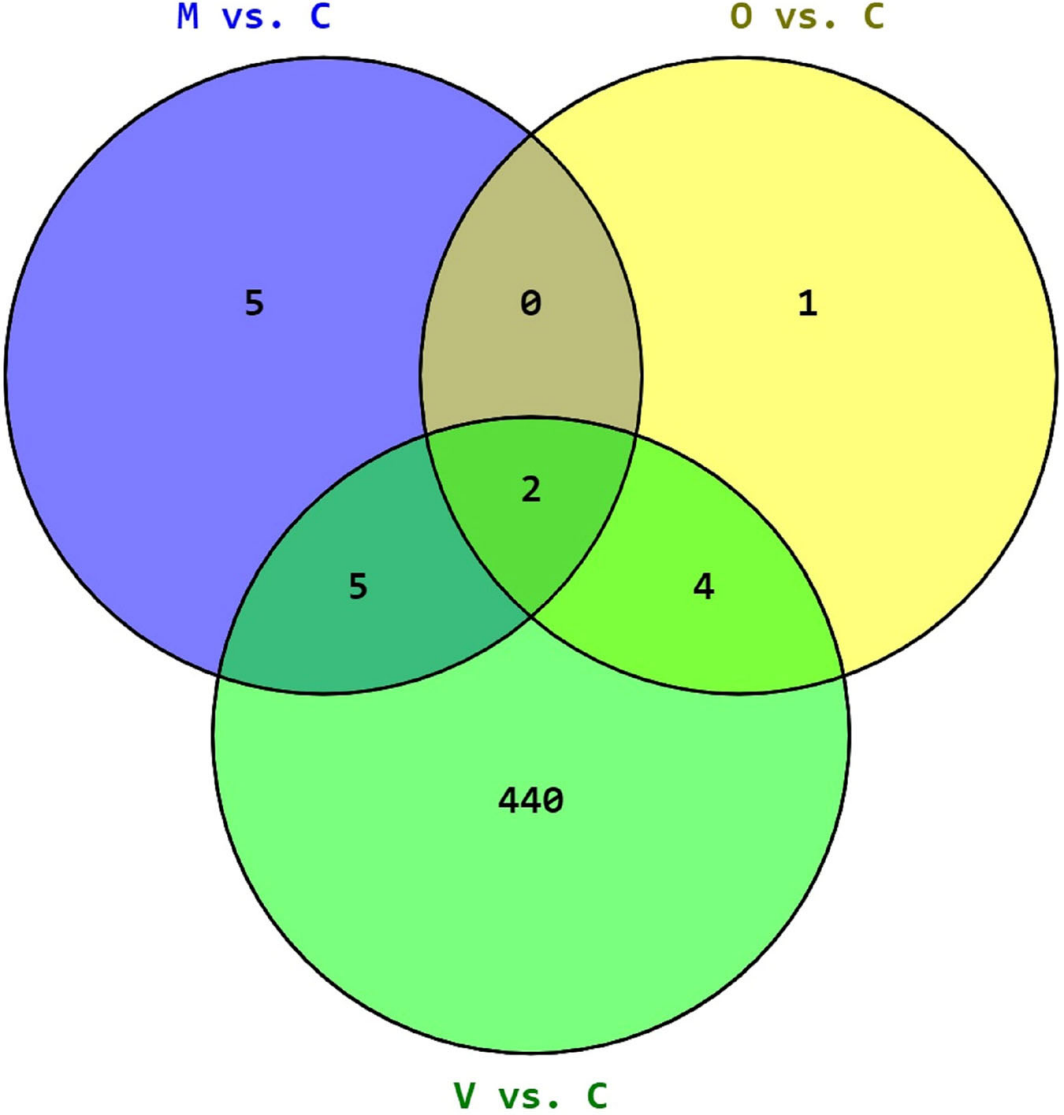
The Venn analysis diagram of the class comparison analyses between the BS patient subgroups and the control group. M versus C ∩ O versus C: *CLEC12A*, *IFI27*; M versus C ∩ V versus C: *CLEC12A*, *IFI27*, *NAPSB*, *EIF1AY*, *XIST*, *TMEM158*, *LRRN3*; O versus C ∩ V versus C: *CLEC12A*, *IFI27*, *FKBP5*, *CDKN1C*, *CLC*, *FECH*; M versus C ∩ O versus C ∩ V versus C: *CLEC12A*, *IFI27*. C, control group; M, mucocutaneous Behçet syndrome group; O, ocular Behçet syndrome group; V, vascular Behçet syndrome group.

The Venn analysis diagram of the DEG sets obtained during class comparison analyses between the BS patient subgroups and the control group is shown in Figure 4.

### 3.5 Findings of the clustering analyses of BS patient subgroups

**Figure 5:**
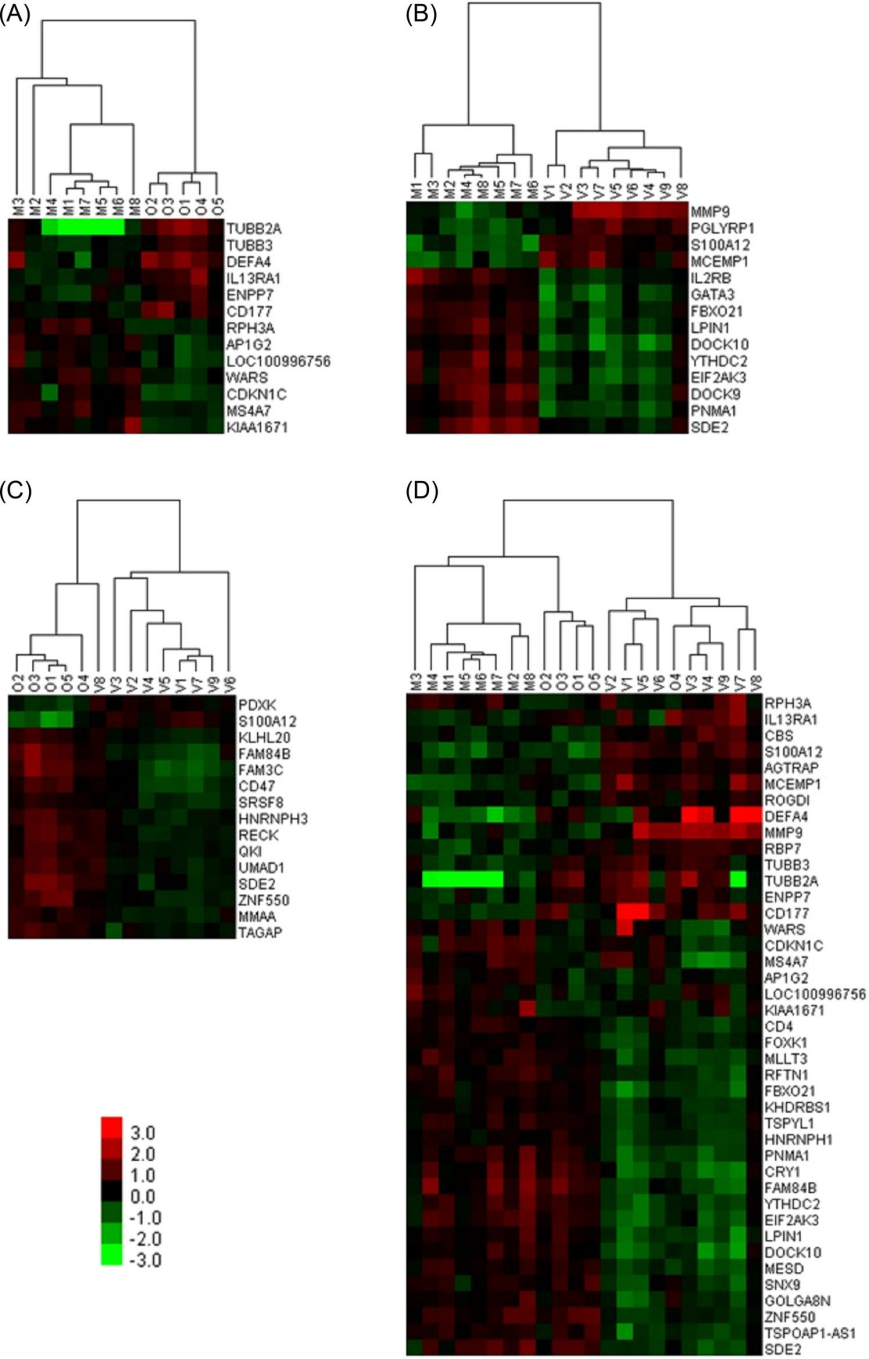
Dendrogram and heatmap representations of clustering analyses performed among Behçet syndrome patient subgroups. (A) M and O, (B) M and V, (C) O and V, and (D) M and O and V. Samples are presented in columns and genes in rows. M1−M8, mucocutaneous Behçet syndrome patients; O1−O5, ocular Behçet syndrome patients; V1−V9, vascular Behçet syndrome patients.

The dendrogram and heatmap representations of clustering analyses performed among subgroups of patients with BS are presented in Figure 5. The clustering algorithm implemented, successfully clustered the BS cases in nearly perfect agreement with their clinical phenotypes. Complete lists of the DEG sets used during clustering analyses can be found in the Supporting Information: 6 File.

### 3.6 Findings of the GO enrichment analyses

Selected findings of the GO enrichment analyses, performed using the DEG sets of the class comparisons M versus C, O versus C, and V versus C, are presented in Table 7 (details regarding the GO term enrichment analyses are given in Supporting Information: 7 File). While innate immunity and hemostasis‐related biological processes were significantly enriched in the M group, adaptive immunity‐specific biological processes were more prominently enriched in the O and V groups.

**Table 7:** Findings of the gene ontology enrichment analyses.^a^ ^,^ ^b^ ^,^ ^c^

GO term	Gene set	Size	Obs	Expect	Ratio	*p*	FDR
M versus C							
Regulation of protein homodimerization activity	0043496	21	2	0.06	34.90	0.00148	0.87182
Type I interferon signaling pathway	0060337	84	3	0.23	13.09	0.00155	0.87182
Cellular response to type I interferon	0071357	84	3	0.23	13.09	0.00155	0.87182
Response to type I interferon	0034340	89	3	0.24	12.35	0.00183	0.87182
Blood coagulation	0007596	318	5	0.87	5.76	0.00167	0.87182
Coagulation	0050817	322	5	0.88	5.69	0.00176	0.87182
Hemostasis	0007599	323	5	0.88	5.67	0.00178	0.87182
Positive regulation of cell migration	0030335	467	6	1.27	4.71	0.00158	0.87182
Response to wounding	0009611	635	7	1.73	4.04	0.00152	0.87182
O versus C							
Regulation of interleukin‐2 production	0032663	54	4	0.27	14.96	0.00015	0.07457
Cellular defense response	0006968	55	4	0.27	14.69	0.00016	0.07457
Regulation of antigen receptor‐mediated signaling pathway	0050854	57	4	0.28	14.18	0.00018	0.07914
T cell‐mediated immunity	0002456	99	5	0.49	10.20	0.00013	0.07352
Regulation of leukocyte migration	0002685	179	7	0.89	7.90	0.00003	0.03764
Myeloid leukocyte migration	0097529	194	7	0.96	7.29	0.00005	0.05503
Lymphocyte mediated immunity	0002449	233	7	1.15	6.07	0.00015	0.07457
Leukocyte cell−cell adhesion	0007159	315	9	1.56	5.77	0.00002	0.03764
Leukocyte migration	0050900	414	11	2.05	5.37	0.00001	0.02614
Adaptive immune response	0002250	377	10	1.87	5.36	0.00002	0.03764
V versus C							
Neutrophil‐mediated killing of symbiont cell	0070943	8	5	1.07	4.66	0.00170	0.03465
Regulation of antigen receptor‐mediated signaling pathway	0050854	57	21	7.64	2.75	0.00001	0.00039
T cell selection	0045058	48	16	6.44	2.49	0.00034	0.00946
Somatic recombination of immunoglobulin gene segments	0016447	49	16	6.57	2.43	0.00044	0.01162
Regulation of interleukin‐12 production	0032655	51	16	6.84	2.34	0.00072	0.01696
Regulation of B cell activation	0050864	111	32	14.89	2.15	0.00001	0.00069
Alpha−beta T cell differentiation	0046632	99	27	13.28	2.03	0.00019	0.00601
Lymphocyte differentiation	0030098	336	89	45.06	1.98	0.00000	0.00000
Regulation of tumor necrosis factor superfamily cytokine production	1903555	134	33	17.97	1.84	0.00032	0.00939
Lymphocyte activation	0046649	636	153	85.30	1.79	0.00000	0.00000

Abbreviations: C, control group; Expect, the expected number of genes to be present in the DEG set, which also belong to the related GO term gene set; FDR, false discovery rate; GO, gene ontology; M, mucocutaneous Behçet syndrome group; O, ocular Behçet syndrome group; Obs, the number of genes common to both the DEG set and the GO term gene set; Ratio, the enrichment ratio; Size, the number of reference genes in the GO term; V, vascular Behçet syndrome group.

^a^ For all class comparisons (M versus C, O versus C, and V versus C), *p* ≤ 0.05 and FC ≥1.5.

^b^ For every class comparison, the top 10 GO terms with immunopathological significance are listed in descending order of their enrichment ratio (ratio) values.

^c^ Enrichment analyses of the GO terms have focused explicitly on the subontology of “Biological Process.”

### 3.7 Findings of the validation experiments

The findings of the validation experiments, performed using a qRT‐PCR approach, are presented in Table 8. For the selected 12 genes, the qRT‐PCR findings of the validation experiments performed were consistent with the microarray findings. Not only the direction of change, but also the amplitude of change were compatible in many instances (Table 8).

**Table 8:** Findings of the qRT‐PCR validation experiments.^a^ ^,^ ^b^

Genes	Class comparison	Microarray FC	qRT‐PCR FC	Comments
*IFI27*	M versus C	3.49	3.83	In accordance (increased expression)
*RSAD2*	M versus C	3.33	2.57	In accordance (increased expression)
*IFI44L*	M versus C	2.94	2.25	In accordance (increased expression)
*CLEC12A*	M versus C	−3.17	0.45	In accordance (decreased expression)
*ARG1*	V versus C	6.55	4.53	In accordance (increased expression)
*DEFA4*	V versus C	5.29	3.97	In accordance (increased expression)
*MS4A1*	V versus C	−6.14	0.26	In accordance (decreased expression)
*IGHM*	V versus C	−5.17	0.29	In accordance (decreased expression)
*IFIT1*	M versus V	3.35	2.68	In accordance (increased expression)
*CMPK2*	M versus V	2.85	2.60	In accordance (increased expression)
*TUBB2A*	M versus V	−6.25	0.53	In accordance (decreased expression)
*ORM1*	M versus V	−2.86	0.76	In accordance (decreased expression)

Abbreviations: C, control group; FC, fold change; M, mucocutaneous Behçet syndrome group; qRT‐PCR, quantitative real‐time polymerase chain reaction; V, vascular Behçet syndrome group.

^a^ The “Delta‐Delta‐Ct” algorithm was used to calculate the qRT‐PCR based expression levels.

^b^ The “housekeeping” gene was selected as Peptidylprolyl Isomerase B (*PPIB*).

## 4 DISCUSSION

This study performed a comparative genome‐wide expression analysis of Turkish BS patients, using a novel study design that differentiated it from other similar studies in the relevant literature.^7^, ^20^, ^21^, ^22^, ^23^, ^24^ Based on the significant differences in gene expression profiles and molecular disease mechanisms of BS subgroups which were previously documented by Oguz et al.,^5^ the present study examined BS patients by grouping them into clusters dictated by their clinical phenotypes. The findings of the study pointed to (1) possible immunopathogenetic roles of the immune response genes *CLEC12A*, *IFI27*, and *CLC* in Turkish BS cases, (2) the presence of significant gene expression differences among distinct BS subgroups, (3) divergent roles and variant predominance of the innate and adaptive immune responses in mucocutaneous versus ocular & vascular BS cases, and (4) a loss of potentially important information at the molecular level by examination of distinct BS clinical phenotypes gathered into a single patient group.

At the beginning of the discussion and before going deep in dissection of the findings, the authors of the study emphasize that the approach of collecting and studying distinct BS clinical phenotypes as a single group, may unintentionally lead to loss of important information, especially at the molecular level. In other words, as the authors we believe that, the study design has a significant impact on the findings of BS studies.

The combined gene‐expression profiling and genome‐wide association study by Xavier et al. is one of the earliest research performed in cases of BS.^7^ According to their findings, Xavier et al. stated that, *EREG*‐*AREG* and *NRG1* genes and the EGF/ErbB signaling pathway might play a role in BS susceptibility.^7^ By borrowing the genome‐wide expression data of the study by Xavier et al. (GEO series GSE17114). Oguz et al. performed a comparative transcriptomic analysis by implementing a novel study design.^5^ The single group of BS patients of the study by Xavier et al. was divided by Oguz et al. into mucocutaneous, ocular, and vascular BS subgroups, according to the patients' clinical phenotypes.^5^, ^7^ Strikingly, this novel study design produced disparate findings. The findings of the study by Oguz et al. can be summarized as; (1) BS patients demonstrate distinct expression profiles in distinct disease subsets, (2) different molecular disease mechanisms seem to be functional in different disease expressions of BS, and (3) four functionally related gene groups, namely, negative regulators of inflammation (*CD69*, *CLEC12A*, *CLEC12B*, *TNFAIP3*), neutrophil granule proteins (*LTF*, *OLFM4*, *AZU1*, *MMP8*, *DEFA4*, *CAMP*), antigen processing and presentation proteins (*CTSS*, *ERAP1*), and regulators of immune response (*LGALS2*, *BCL10*, *ITCH*, *CEACAM8*, *CD36*, *IL8*, *CCL4*, *EREG*, *NFKBIZ*, *CCR2*, *CD180*, *KLRC4*, *NFAT5*) appear to be instrumental in BS immunopathogenesis.^5^ Based on their findings, Oguz et al. concluded that, the designation as “Behçet syndrome” should be encouraged and future research should take into consideration the immunogenetic heterogeneity of BS clinical phenotypes.^5^ Interestingly, *CLEC12A* which appeared in the “negative regulators of inflammation” gene group of Oguz et al., was also a significant finding of the present study.^5^

Two innate immune response genes drew special attention, namely *CLEC12A* and *IFI27* in the DEG sets of the class comparisons M versus C, O versus C, and V versus C. Besides their potential functional significance in BS pathogenesis, *CLEC12A* and *IFI27* both were among the top 10 DEGs (Table 6). Table 9 presents the FC values of these two genes, obtained by the BS subgroup versus control group class comparisons. Strikingly, also during the Venn analysis, *CLEC12A* and *IFI27* were found to be the only two genes present in the intersection M versus C ∩ O versus C ∩ V versus C (Figure 4).

**Table 9:** The fold change values of *CLEC12A* and *IFI27* genes in BS subgroup versus control group class comparisons.

Gene	Class comparison			Comment
	M versus C	O versus C	V versus C	
*CLEC12A*	−3.17	−2.78	−2.35	Decreased expression in the first class (e.g., M) compared to the second class (e.g., C).
*IFI27*	3.49	2.14	4.34	Increased expression in the first class (e.g., M) compared to the second class (e.g., C).

Abbreviations: C, control group; M, mucocutaneous Behçet syndrome group; O, ocular Behçet syndrome group; V, vascular Behçet syndrome group.

*CLEC12A* (C‐type lectin domain family 12 member A, also known as *MICL*, *KLRL1*, *CD371*) is an innate immune system receptor, expressed on the surface of granulocytes, monocytes/macrophages, and NK lymphocytes with an inhibitory function.^25^, ^26^ It appears that *CLEC12A* exerts its inhibitory effect via the immunoreceptor tyrosine‐based inhibitor motif (ITIM) present on its cytoplasmic tail.^25^, ^26^ *CLEC12A*, of which uric acid crystals have been shown as one of its endogenous ligands, is thought to have an important role in regulating the immune response and maintaining homeostasis by reducing the severity of the inflammatory reaction, especially in the presence of tissue injury.^27^, ^28^, ^29^ As an important finding, it has been reported that the anti‐inflammatory agent colchicine, which is currently prescribed for the treatment of BS, gout, and Familial Mediterranean Fever all of which are neutrophil‐mediated inflammatory diseases, induces the expression of *CLEC12A*.^30^ Studies of rheumatoid arthritis (RA) have also shown that, loss of *CLEC12A* function or its decreased expression is associated with an increased RA disease activity and inflammation severity.^31^, ^32^, ^33^ Oguz et al., after noticing the decreased expression of *CLEC12A* in Turkish BS cases during preliminary analysis of their transcriptome data and by collecting the findings in the literature on *CLEC12A*, proposed the hypothesis that *CLEC12A* may be a common denominator in the development of BS and gout.^34^ In support of their hypothesis, Oguz et al. pointed to the findings of (1) negative correlation of *CLEC12A* expression with hyperinflammatory responses, (2) the presence of *CLEC12A* polymorphisms with functional and clinical effects in certain inflammatory diseases, (3) the dual use of colchicine for the treatment of BS and gout, (4) the exaggerated inflammatory response to uric acid crystals detected in both BS and gout cases, (5) the presence of the genomic locus of the *CLEC12A* gene (i.e., 12p12‐13), among the findings of the GWAS and GWLS of BS, and (6) their preliminary finding of decreased *CLEC12A* expression in Turkish BS cases.^34^ At the end of their hypothesis article, Oğuz et al. stated that, if their hypothesis about *CLEC12A* is proved with well‐designed studies in the future, scientists may be able to go a long way toward the elucidation of the pathogenesis of BS & gout, and also an animal model development for BS.^34^ In a more recent review, French researcher Elise Chiffoleau emphasized that, C‐type lectin‐like receptors, including *CLEC12A*, could be potential treatment targets by playing important roles in the regulation of sterile inflammation.^35^ Another recent research by Paré et al. documented the early molecular events by which *CLEC12A* inhibit neutrophil activation and cytokine release.^36^ An important point to emphasize here is, *CLEC12A* ranked among the top 10 DEGs of the class comparisons M versus C and O versus C. When the Y chromosome genes (interfering as a result of the male/female ratio discrepancy of the compared classes) are excluded from the M versus C list, *CLEC12A* becomes the most downregulated gene in both of the M versus C and O versus C class comparisons (Table 6). We believe that, all of these findings presented in a summarized manner support that, decreased expression of *CLEC12A*, which is a common finding in different clinical phenotype clusters of Turkish BS patients may have a role in the development of already well‐documented exaggerated neutrophil functions and hyperinflammatory innate immune response in BS patients.

*IFI27* (interferon alpha inducible protein 27, also known as *P27*, *ISG12*, *FAM14D*) is one of the genes belonging to the group of interferon‐stimulated genes (ISG), mediating the antiviral, immunomodulatory, and antiproliferative effects of interferons.^37^ During the years following their first description, interferon molecules were believed to be an important component of the innate immune system and its response against viruses due to their powerful antiviral effects. Today, interferons and ISGs that mediate their biological effects, are considered to function in both innate and adaptive arms of the immune system, and are thought to have important roles in the development of the close communication and cooperation between these two inseparable components of the immune system.^38^, ^39^ The literature harbors many inflammatory/immunological conditions including inflammatory bowel diseases, psoriasis, systemic lupus erythematosus, Sjögren's syndrome, antiphospholipid syndrome, acute graft‐versus‐host disease, immune thrombocytopenic purpura, Aicardi−Goutieres syndrome, Kikuchi−Fujimoto, and hand, foot, and mouth diseases, in which significant increases in *IFI27* expression have been reported.^39^, ^40^, ^41^, ^42^, ^43^, ^44^, ^45^, ^46^, ^47^, ^48^, ^49^, ^50^ It is also well‐known that, some of these above‐mentioned inflammatory diseases (i.e., inflammatory bowel diseases, psoriasis, and systemic lupus erythematosus) have an intersection with BS.^51^, ^52^, ^53^ Regarding interferons in BS, Belguendouz et al. reported an increased in vivo and in vitro production of interferon gamma in active ocular BS patients compared to inactive patients and healthy controls.^54^ Additionally in the same study, interferon gamma was shown to induce nitric oxide production in vitro.^54^ Based on their findings, Belguendouz et al. concluded that, interferon gamma was implicated in the occurrence of the inflammatory process of Behçet uveitis.^54^ Similar to *CLEC12A*, *IFI27* is also among the top 10 most DEG of the class comparisons (second in M vs. C with an FC: 3.49, second in O vs. C with an FC: 2.14, and third in V vs. C with an FC: 4.34) (Table 6). As a remarkable and contradictory finding of the genome‐wide expression study of BS by Okuzaki et al., *IFI27* was reported to be among the DEGs with a decreased expression in the patient (i.e., BS) group.^21^ This contrasting situation may be noted as another good example of the discordant immunological findings observed in BS studies. Even though the association of increased *IFI27* expression with inflammatory conditions has been consistently documented, the mechanisms underlying this finding remain to be clarified (i.e., whether *IFI27* has a direct role here or *IFI27* merely represents the presence of an innate immune proinflammatory cytokine response including the interferons).

Another gene that drew attention in the class comparisons of the ocular and vascular BS groups with the control group was *CLC*. As is presented in Table 6, *CLC* ranked second with an FC of −2.57 for O versus C and 7th with an FC of −3.57 for V versus C class comparisons. *CLC* (Charcot−Leyden crystal protein, also known as *GAL10*, *LGALS10*) has historically been identified as an important component of human eosinophil leukocytes and is named after the Charcot−Leyden crystals, which are frequently observed at sites of eosinophilic inflammation.^55^ *CLC*, which was thought to have lysophospholipase function for a long period of time, is now accepted as a member of the galectin (lectin) gene family.^56^ *CLC*, with a primary carbohydrate affinity for mannose, is shown to play a role in immune system surveillance against inflammation and tumors.^57^, ^58^ A well‐designed study by Kubach et al. revealed a very important new function for *CLC* that came as a surprise.^59^ According to the study findings of Kubach et al., *CLC* is highly expressed in CD4^+^CD25^+^ regulatory T cell (Treg cell) cytoplasm, and this expression seems to be essential for the adaptive immune response suppressive functions of the regulatory T lymphocytes.^59^ In the same study, specific inhibition of the *CLC* protein in regulatory T lymphocyte cytoplasm resulted in loss of regulatory T lymphocyte suppressor function.^59^ In a more recent study, Lingblom et al. showed that CD16^+^ (high expression) eosinophil granulocyte population suppressed T lymphocyte functions via *CLC*.^60^ Taken together with these literature information, our findings regarding decreased expression of *CLC* in the ocular and vascular BS subgroups may indicate that, in addition to *CLEC12A* and *IFI27*, *CLC* may also be playing a role in (1) the emergence of the characteristic hyperinflammatory manifestations of BS, and (2) the addition of the adaptive immune response to the initial scene of innate immunity in BS, particularly in ocular and vascular cases.

Mucocutaneous findings (i.e., recurrent oral and genital aphthae, various inflammatory skin lesions, and positive pathergy test) are hallmarks of BS which unequivocally occur in BS cases, regardless of their disease clusters.^61^ This strongly raises the possibility of a common/shared pathogenetic component among the BS disease clusters. Venn analysis performed on the DEG lists of the M versus C, O versus C, and V versus C class comparison analyses of our study displayed that, the intersection set of these three lists contained the two genes, *CLEC12A* and *IFI27*, which are already discussed (Figure 4). When we consider that the oral and genital areas and the skin have their unique and heavily crowded microbial floras, and the microbial breaches occurring at these sites, are initially and primarily confronted with components of the innate immune system (e.g., neutrophil granulocytes), the presence of the genes *CLEC12A* and *IFI27*, in the intersection of M versus C, O versus C, and V versus C class comparisons, makes good sense. Oguz et al. performed a similar Venn analysis in their study and interestingly found the same intersection set to be empty (i.e., none shared DEG between M vs. C, O vs. C, and V vs. C class comparisons).^5^ Oguz et al. presented this finding as an important evidence of the heterogeneity at the molecular level among BS disease clusters.^5^

The gene set enrichment analyses of our study yielded distinct results in different BS subgroups. As can be seen in Table 7, GO terms titled “Type I interferon signaling pathway,” “Coagulation,” and “Response to injury” gained special importance in mucocutaneous BS cases. “Type I interferon signaling pathway” and “Response to injury” categories are essentially innate immunity‐related titles. What is striking here is that in mucocutaneous BS cases, who are accepted to be at the “mild“ end of the BS disease spectrum, any processes related to the adaptive immunity did not show significant enrichment. As previously mentioned, today, BS is accepted as a complex inflammatory condition which is triggered by an exaggerated/aberrant innate immune response and is sustained by adaptive immune responses.^62^ Consistent with our interferon (type I, specifically interferon alpha) signaling pathway finding, interferon signal activation were also reported in two previous BS studies by Puccetti et al. and Tulunay et al.^20^, ^22^ Coagulation was another significant GO term which drew attention and deserved a mention in the mucocutaneous BS subgroup. Puccetti et al. also showed the “Coagulation” category among the enriched GO categories in their study.^22^ Although the prothrombotic state observed in cases of BS has been well‐described in the literature, the molecular mechanisms responsible for this occurrence still await clarification.^63^ Currently, neutrophil leukocytes' ability of “neutrophil extracellular trap” (NET) generation and thereby thrombosis formation, is increasingly emphasized in the BS literature.^64^, ^65^ When the genes which are both present in the DEG sets and also in the GO terms with the titles “Coagulation,“ “Blood coagulation,“ and “Hemostasis“ are examined, *C1QBP*, *F13A1*, *H3F3A*, *ITGA2B*, and *TREML1* (in alphabetical order) are noticed (Supporting Information: 7 File). At least three of these genes (i.e., *H3F3A*, *ITGA2B*, and *TREML1*) are currently reported to be among the genes involved in NET formation.^66^, ^67^ Even in the case of mucocutaneous BS cases, who are thought to be at the “mild” end of the BS disease spectrum, we believe that, the presence of findings related to NET formation and accompanying “immunothrombosis” may shed light on the development of the well‐known prothrombotic state of BS. Finally, another GO term that showed enrichment in our mucocutaneous BS subgroup was “Response to injury.” We believe that, the frequent and recurrently occurrence of mucosal surface and skin lesions in mucocutaneous BS cases makes this finding reasonable.

When gene set enrichment analyses of the ocular and vascular BS subgroups are reviewed, in addition to the GO terms such as “Immune response,“ “Leukocyte migration,“ and “Leukocyte activation“ which refer to immune responses in general, GO terms such as “Adaptive immune response,“ “Lymphocyte activation,“ “IL‐2 production,” “Lymphocyte differentiation,” and “T cell mediated immunity” which are specifically related to the adaptive immune response were found to show significant enrichment (Table 7). In the case of ocular and vascular BS, which are thought to be at the “severe” end of the BS disease spectrum, tissue damage occurs more severely and in a long standing manner, which may cause display of novel antigenic determinants from these injured “self” tissues, and thereby stimulate the patients' adaptive immune system further.^68^, ^69^, ^70^, ^71^, ^72^ In three other genome wide‐expression studies of BS by Tulunay et al., Okuzaki et al., and Puccetti et al., findings supporting the contribution of an adaptive immune response were also evident.^20^, ^21^, ^22^

The findings of the gene set enrichment analyses of our study which we briefly mentioned above, can be wrapped up as “an immune‐mediated disorder with innate and adaptive immune responses contributing to it, which displays an innate immunity predominance in its “mild” forms, whereas adaptive immunity is in front in its “severe” forms.” This statement seems to be in harmony with the current pathogenetic mechanism definition of BS which goes as follows: “A complex genetic background leading to a proinflammatory, innate‐immune system derived activation perpetuated by adaptive immune responses against environmental and autoantigens.”^62^, ^73^

Like every other scientific study, our study has its own limitations. One of these limitations is about the transcriptome profiling technology implemented in our research. While transcriptome profiling can be performed by hybridization or next‐generation sequencing based (RNA‐seq) methodologies and we have implemented a microarray, therefore a hybridization based methodology, RNA‐seq has well known advantages when compared with microarrays. Just to make a brief mention of these advantages, we should point that, RNA‐seq enables detection of biologically relevant genetic variants (e.g., any gene fusions, single nucleotide variants including polymorphisms or mutations, indels, alternatively spliced transcripts, and specifically, functionally different isoforms), quantification of gene expression across a wider dynamic range with absolute values, performing analysis with a low total RNA, obtaining larger DEG sets with a higher sensitivity, and identification of the rare and low‐abundance transcripts compared to microarray experiments. There are also a few points to mention, regarding the design of the research. First and foremost, it would be a better practice to view and interpret our findings while bearing in mind that they belong to a Turkish BS population. Because of major ethical concerns related to the potential threatening consequences of discontinuing their therapeutic schemes, every BS case enrolled in our study continued his/her pharmacological therapy, and this should be noted as another limitation of our study. When contrasted with similar other studies in the literature, the total number of BS cases present in our study is seemingly high. Nevertheless, the lack of BS patients with involvements of relatively uncommon organ systems (i.e., gastrointestinal, central nervous, and musculoskeletal systems) may be listed as an additional limitation of our study. Thus, we believe that it is important to plan and conduct new studies with large numbers of treatment‐naive BS cases. Still another important point to remember is that, although we have searched for gene expression differences observed in BS patients, we did not obtain any information about the mechanisms responsible for the observed gene expression differences. Both epigenetic control, in the form of DNA methylation or histone modification variations, and certain single nucleotide polymorphisms (SNPs), located in the upstream and downstream transcriptional control sequences of the respective genes may well be responsible for the observed expression differences. This situation requires planning for further epigenetic or SNP (expression quantitative trait loci) analyses, which will seek to document epigenetic and genetic variants that affect the expression levels of these genes. It is also essential that the findings of our study should be validated in another Turkish BS patient cohort.

A supplement to “Discussion” section can be found in Supporting Information: 8 File.

## 5 CONCLUSION

This comparative gene‐expression profiling study of Turkish BS cases revealed a couple of important findings regarding the immunopathogenesis of BS. First of all, three genes, *CLEC12A*, *IFI27*, and *CLC*, appeared to be potentially instrumental in BS immunopathogenesis and these genes may be of value for therapeutic targeting and animal model development purposes. The authors of the study believe that, at least in the case of Turkish BS patients, novel targeted therapy drugs specifically “targeting” *CLEC12A*, *CLC*, and *IFI27* genes/proteins may prove to be of value for therapeutic purposes. It was also shown that BS patients displayed distinct gene expression profiles and molecular disease mechanisms in different BS clinical phenotypes. A significant consequence of this finding appeared as a loss of information with the single group analysis of BS patients of different disease clusters. The authors believe that the nomenclature as the “Behçet syndrome” should be preferentially utilized and future research should take into account the molecular level heterogeneity of the distinct BS disease clusters. New studies enrolling treatment naive BS patients will be of great value and will add vital information to the topic.

## AUTHOR CONTRIBUTIONS

Mehmet Nejat Akar and Ali Kemal Oğuz conceived and designed the research. Ali Kemal Oğuz collected the data (and the samples). Hilal Özdağ, Ali Kemal Oğuz, and Seda Taşır performed the analysis (the in vitro experiments). Hilal Özdağ, Ali Kemal Oğuz, and Seda Taşır analyzed the data (the bioinformatics). Ali Kemal Oğuz and Çağdaş Şahap Oygür wrote the manuscript. Ali Kemal Oğuz, Çağdaş Şahap Oygür, Seda Taşır, Hilal Özdağ, and Mehmet Nejat Akar all have read and approved the final submitted version of the manuscript.

## CONFLICT OF INTEREST STATEMENT

The authors declare no conflict of interest.

## ETHICS STATEMENT

The Ethics Committee of Ufuk University (Ankara, Turkey) approved the study with decision number 08065 dated 06.24.2009.

## Supporting information


**Support1 File**.

**Support2 Fig**.

**Support3 File**.

**Support4 File**.

**Support5 File**.

**Support6 File**.

**Support7 File**.

**Support8 File**.

## Data Availability

The data that support the findings of this study are openly available in GEO at https://www.ncbi.nlm.nih.gov/geo/query/acc.cgi?acc=GSE209567, reference number GSE209567. The metadata, raw, and final/normalized data of the study are deposited to Gene Expression Omnibus repository (accession: GSE209567).
