# Supplementary material for: Behçet syndrome: The disturbed balance between anti‐ (CLEC12A, CLC) and proinflammatory (IFI27) gene expressions
Source: Immun Inflamm Dis. 2023 Apr 12;11(4):e836. doi: 10.1002/iid3.836 (PMC10091377; doi:10.1002/iid3.836)
Supplement: Supplementary file 1 — Support1 File. [file IID3-11-e836-s006.doc]

**COMPARATIVE TRANSCRIPTOMIC ANALYSIS**

**IN PATIENTS WITH BEHÇET SYNDROME**

**CASE REPORT FORM**

**PATIENT NAME (INITIALS):** **DATE:** / / 201

**GENDER:** M [ ] F [ ] **AGE:** **TELEPHONE:**

**AGE OF ONSET OF BEHÇET’S SYNDROME:**

**ORAL ULCER:** (+) [ ] **GENITAL ULCER:** (+) [ ] (-) [ ]

**PATHERGY:** (+) [ ] (-) [ ] **SKIN LESIONS:** (+) [ ] (-) [ ]

**OCULAR INVOLVEMENT:** (+) [ ] (-) [ ]

**CNS INVOLVEMENT:** (+) [ ] (-) [ ] **GIS INVOLVEMENT:** (+) [ ] (-) [ ]

**CARDIAC INVOLVEMENT:** (+) [ ] (-) [ ] **PULMONARY INVOL.:** (+) [ ] (-) [ ]

**EPIDIDYMITIS:** (+) [ ] (-) [ ] **MUSCULOSKE. INVOL.:** (+) [ ] (-) [ ]

**VASCULAR INVOLVEMENT:** (+) [ ] (-) [ ]

**DVT:** [ ] **VCS:** [ ] **VCI:** [ ] **HEPATIC / PORTAL VEIN:** [ ] **AORT:** [ ]

**ANY EXACERBATIONS DURING THE LAST 2 MONTHS PRECEDING THE**

**EVALUATION EXCLUDING ORAL ULCERS:** (+) [ ] (-) [ ]

**MEDICATIONS USED AT THE TIME OF EVALUATION**

**COLCHICINE:** [ ]

**GLUCOCORTICOID:** [ ] **NSAID:** [ ]

**AZA:** [ ] **CYCLOSPORINE:** [ ] **CYC:** [ ] **CHLORAMBUCIL:** [ ] **MTX:** [ ]

**IFN:** [ ] **SULFASALAZINE:** [ ]

**ASA:** [ ] **DIPYRIDAMOLE:** [ ] **CLOPIDOGREL:** [ ] **LMWH:** [ ] **WARFARIN:** [ ]

**TNF INHIBITOR:** [ ] **ETANERCEPT:** [ ] **INFLIXIMAB:** [ ] **ADALIMUMAB:** [ ]

**OTHER (PLEASE SPECIFY):**

**THANK YOU VERY MUCH FOR YOUR CONTRIBUTION**

**M1 / O1 / V1 / D1 / C1 ← Please Write on Each of the Two Tubes**

**Please Fill the Tubes Completely and Mix!**
