# Supplementary figures and images for: Behçet syndrome: The disturbed balance between anti‐ (CLEC12A, CLC) and proinflammatory (IFI27) gene expressions
Source: Immun Inflamm Dis. 2023 Apr 12;11(4):e836. doi: 10.1002/iid3.836 (PMC10091377; doi:10.1002/iid3.836)

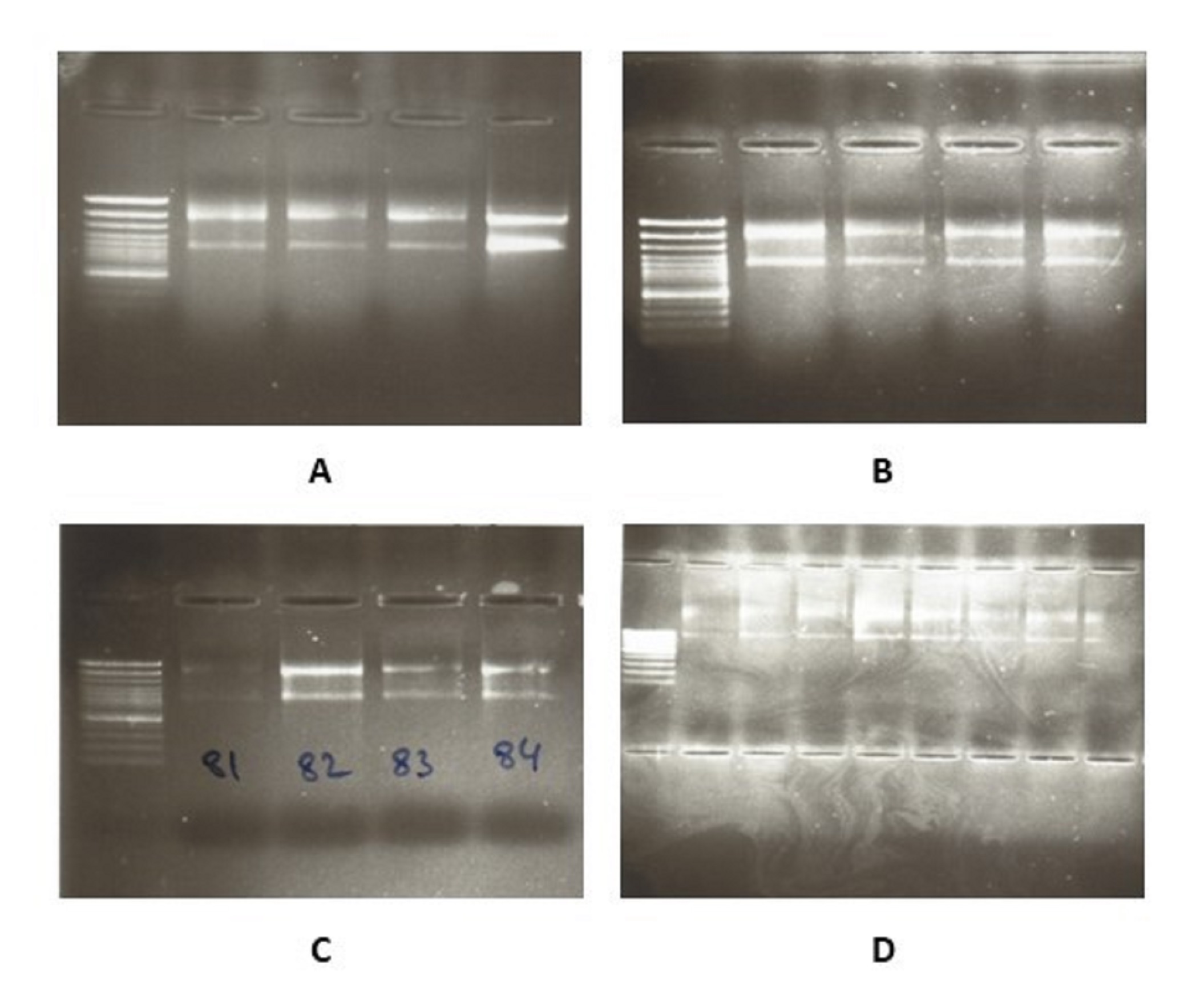

Supplement: Supplementary file 2 — Support2 Fig. [file IID3-11-e836-s002.jpg]
