# Supplementary material for: Behçet syndrome: The disturbed balance between anti‐ (CLEC12A, CLC) and proinflammatory (IFI27) gene expressions
Source: Immun Inflamm Dis. 2023 Apr 12;11(4):e836. doi: 10.1002/iid3.836 (PMC10091377; doi:10.1002/iid3.836)
Supplement: Supplementary file 8 — Support8 File. [file IID3-11-e836-s004.docx]

# Supplement to Discussion

A careful search of the literature revealed six studies, including works of Xavier et al., Tulunay et al., Okuzaki et al., Puccetti et al., Verrou et al., and Zheng et al., which performed genome-wide expression analyses in BS.^1–6^ Important information regarding these studies is summarized in Table S1. Although BS is currently accepted as a syndrome and there is a good deal of evidence supporting this approach, the clinical and molecular studies of BS almost without exception, gather and examine BS cases as a single group of patients despite the remarkable presence of distinct clinical phenotypes among these patients.^1–8^ As can be seen below in Table S1, these six studies are no exception and they all have collected and studied the BS cases enrolled, as a single patient group. Also, there are two important points that should be emphasized regarding these studies. First of all, every one of these six studies belong to a different country and therefore represent BS cases of different populations with different characteristics (i.e., Xavier et al. Portugal, Tulunay et al. Turkey, Okuzaki et al. Japan, Puccetti et al. Italy, Verrou et al. Greece, and Zheng et al. China).^1–6^ It is worth remembering that, even along the “Silk Road” which is accepted as the characteristic BS geography, there appear to be prominent regional differences regarding the clinical manifestations of BS.^9^ Today, BS is essentially considered a complex autoinflammatory condition, and in a similar manner to other complex diseases, distinct immunogenetic characteristics of the relevant population as well as the locally effective environmental factors may be instrumental in BS pathogenesis.^10^ The second point which deserves special attention is about the findings of these six studies, which clearly demonstrate conflicting as well as overlapping features. One example of this situation may be the finding of Puccetti et al., which indicates an autoimmune component in the pathogenesis of BS, against the finding of Verrou et al., which states that there is no adaptive immune response component in BS pathogenesis.^4,5^ As previously stated, the literature on BS harbors divergent and sometimes contrasting immunological findings among BS cases and studies. In order to clarify these divergent and sometimes conflicting immunological aberrations observed in BS patients, four fundamental explanations were put forth; (1) varying clinical activity of BS patients among different studies, (2) inconsistency between the "in-vitro" findings and the "in-vivo" state, (3) hard to standardize differential drug effects, and (4) results pertaining to different laboratory methodologies.^11^ Currently, in addition to above-mentioned factors, molecular level variations observed among BS cases and BS clinical phenotypes may be listed as another explanatory factor, for the inconsistent immunological aberrations identified in BS patients.^12^

**Table S1. Important information regarding gene-expression profiling studies of Behçet syndrome present in the current literature.**

| **Study** | **Year** | **Case/Control**  **Counts** | **Case**  **Groups** | **Method** | **Cell**  **Tissue** | **P/FC** | **Additional**  **Analyses** |
| --- | --- | --- | --- | --- | --- | --- | --- |
| **Xavier et al.**^1^ | 2013 | 15/14 | Single | Microarray | PBMC | 0.05/1.2 | GWAS |
| **Tulunay et al.**^2^ | 2014 | 9/9 | Single | Microarray | M & Th | -/1.5 | FlCy |
| **Okuzaki et al.**^3^ | 2016 | 41/17 | Single | Microarray | PBMC | -/- | Sequencing |
| **Puccetti et al.**^4^ | 2018 | 10/10 | Single | Microarray | PBC | 0.01/2.0 | FlCy |
| **Verrou et al.**^5^ | 2021 | 14/11 | Single | NGS | PBMC | 0.05/2.0 | - |
| **Zheng et al.**^6^ | 2022 | 13/14 | Single | NGS^7^ | PBMC & M | 0.01/1.0 | FlCy/qRT-PCR |

^1^ Xavier et al. 2013.^1^

^2^ Tulunay et al. 2014.^2^

^3^ Okuzaki et al. 2016.^3^

^4^ Puccetti et al. 2018.^4^

^5^ Verrou et al. 2021.^5^

^6^ Zheng et al. 2022.^6^

^7^ Both single-cell and bulk RNA sequencing were performed.

FC, fold change; FlCy, flow cytometry; GWAS, genome-wide association study; M, monocyte; NGS, next-generation sequencing; PBC, peripheral blood cell; PBMC, peripheral blood mononuclear cell; qRT-PCR, quantitative reverse transcription polymerase chain reaction; Th, helper T cell.

As previously stated, among the 29 BS cases that constituted the patient group of our study, 3 BS cases with musculoskeletal involvement (D1, D3, and D7), 1 BS case with gastrointestinal system involvement (D2), 1 another BS case with central nervous system involvement (D6), and 2 BS cases with concurrent ocular and vascular involvements (D4 and D5) were excluded from further analyses, due to the study design and their small sample sizes (Table 4).^13^ Studies dealing with the epidemiology of BS consistently demonstrate an equal/nearly equal male/female ratio and a more severe disease course in male patients on the endemic geography of the syndrome (i.e., Silk Road); while the situation seems to be disparate in geographical regions where BS is rarely observed, such that the number of female cases outweighs males and female BS cases have a milder course of the syndrome.^14,15^ The male/female ratio of 1.2 of the 22 BS cases including the mucocutaneous, ocular, and vascular BS groups and the proportional dominance of females in the mucocutaneous group (1/7) and males in the vascular group (9/-), are in harmony with the relevant literature on the epidemiological characteristics of BS (Table 3).

The number of DEGs obtained during the multiple class comparisons performed between BS patients (B), BS subgroups (M, O, and V), and healthy controls (C) were presented in Table 5. When carefully analyzed, the DEG numbers of B *vs.* C: 28, V *vs.* C: 555, M *vs.* V: 324, and O *vs.* V: 142 (P≥0.05 and FC≥2.0 for all comparisons) point to, (1) the presence of significant discrepancies between BS subgroups regarding gene expressions, and (2) a loss of molecular level information with the single group evaluation approach of distinct BS clinical phenotypes. Identic findings were previously obtained and reported by Oguz et al.^12^ In their paper, Oguz et al. pointed to a marked heterogeneity at the transcriptomic level among distinct BS subgroups and they commented that, the class comparison of all BS cases together as a single group with the control group resulted in a significant reduction of the number of DEGs that otherwise could be revealed.^12^ Accordingly, in the same study, individual comparison of specific BS subgroups/clinical phenotypes with the control group yielded significantly larger numbers of DEGs (i.e., B *vs.* C: 4, M *vs.* C: 5, O *vs.* C: 151, and V *vs.* C: 274, P≥0.05 and FC≥2.0 for all comparisons).^12^ Taken together these two findings clearly indicate that, BS patients display opposite changes in gene expression in different disease clusters. In line with this transcriptome-level finding, contrasting immunological findings had previously been reported in the literature on BS (e.g., increased, normal or decreased neutrophil functions).^16^

It would be important to mention the analogy between the *CD69* and *TNFAIP3* genes pointed out in the study by Oguz et al., and the *CLEC12A* and *CLC* genes which came forward in our study.^12^ *CD69* (also known as *MLR-3*, *CLEC2C*) is another C-type lectin receptor, similar to *CLEC12A* and *CLC*. *CD69*, which was thought to be an activation marker of many leukocytes when it was initially described, has been shown to exert important inflammation suppressive functions in recent studies.^17–19^ A second remarkable mimicry is the genomic locus of *CD69* (i.e., 12p13.31) which it shares with *CLEC12A*. The *TNFAIP3* gene coding for the protein A20, displays a very strong anti-inflammatory effect by inhibiting NF-κB activation.^20^ Zhou et al. showed that A20 haplo-deficiency caused by loss-of-function mutations of the *TNFAIP3* gene leads to a familial, very early (infancy) onset multisystem inflammatory disease with a great resemblance to BS.^21^ In the study of Oguz et al., it was pointed out that the concomitant decreased expressions of *CD69* and *TNFAIP3* genes, which both had important anti-inflammatory functions, was associated with the occurrence of vascular BS, considered to be a severe form of BS.^12^ Similarly, in our study it appeared that, *CLEC12A* and *CLC* genes, both of which display important anti-inflammatory functions, showed decreased expressions together in ocular and vascular BS subgroups (Table 6).

The results of the clustering analyses of our study were in good agreement with the clinical phenotype grouping of the BS cases (Fig 5). The marked success of the clustering, provided an additional evidence of the heterogeneity at the molecular level, among the BS cases belonging to different disease clusters. The explanation for the discordant clustering of the case “V8” together with the ocular BS cases can be made by, the argument that this case has an increased risk of ocular involvement, even though he has not yet experienced any ocular involvement during his disease course until the time of enrollment in the study (Fig 5, panel C). In the combined cluster analysis of all BS cases, this time “O4” was aberrantly clustered together with vascular BS cases (Fig 5, panel D). A similar approach, stating the increased risk of a vascular involvement for “O4” could be made. Another finding worth-mentioning is the close clustering of the mucocutaneous and ocular cases, on the same arm of the clustering dendrogram in Fig 5, panel D. A possible explanation for this finding can be made by the fact that, when compared with vascular BS cases, mucocutaneous and ocular BS cases relatively resemble each other at the molecular level (i.e., the number of DEGs of the class comparisons among the BS subgroups: M *vs.* O: 6, M *vs.* V: 324, and O *vs.* V: 142) (Table 5).

# References (for Supplement to Discussion)

1. Xavier JM, Krug T, Davatchi F, et al. Gene expression profiling and association studies implicate the neuregulin signaling pathway in Behçet’s disease susceptibility. *J Mol Med*. 2013;91(8):1013-1023. doi:10.1007/s00109-013-1022-4

2. Tulunay A, Dozmorov MG, Ture-Ozdemir F, et al. Activation of the JAK/STAT pathway in Behcet’s disease. *Genes Immun*. 2015;16(2):170-175. doi:10.1038/gene.2014.64

3. Okuzaki D, Yoshizaki K, Tanaka T, et al. Microarray and whole-exome sequencing analysis of familial Behçet’s disease patients. *Sci Rep*. 2016;6(1):19456. doi:10.1038/srep19456

4. Puccetti A, Fiore PF, Pelosi A, et al. Gene Expression Profiling in Behcet’s Disease Indicates an Autoimmune Component in the Pathogenesis of the Disease and Opens New Avenues for Targeted Therapy. *J Immunol Res*. 2018;2018:1-18. doi:10.1155/2018/4246965

5. Verrou K-M, Vlachogiannis NI, Ampatziadis-Michailidis G, et al. Distinct transcriptional profile of blood mononuclear cells in Behçet’s disease: insights into the central role of neutrophil chemotaxis. *Rheumatology*. January 2021. doi:10.1093/rheumatology/keab052

6. Zheng W, Wang X, Liu J, et al. Single-cell analyses highlight the proinflammatory contribution of C1q-high monocytes to Behçet’s disease. *Proc Natl Acad Sci*. 2022;119(26). doi:10.1073/pnas.2204289119

7. Bettiol A, Prisco D, Emmi G. Behçet: the syndrome. *Rheumatology (Oxford)*. 2020;59(Suppl 3):iii101-iii107. doi:10.1093/rheumatology/kez626

8. Yazici H. Behçet Syndrome as a Construct. *Turkish J Med Sci*. 2020;50(SI-2):1585-1586. doi:10.3906/sag-2002-145

9. Yurdakul S. Epidemiology of Behçet Syndrome and Regional Differences in Disease Expression. In: *Behçet Syndrome*. Cham: Springer International Publishing; 2020:21-35. doi:10.1007/978-3-030-24131-5_3

10. Gul A. Behçets Disease as an Autoinflammatory Disorder. *Curr Drug Target -Inflammation Allergy*. 2005;4(1):81-83. doi:10.2174/1568010053622894

11. Greco A, De Virgilio A, Ralli M, et al. Behçet’s disease: New insights into pathophysiology, clinical features and treatment options. *Autoimmun Rev*. 2018;17(6):567-575. doi:10.1016/j.autrev.2017.12.006

12. Oğuz AK, Yılmaz ST, Oygür ÇŞ, et al. Behçet’s: A Disease or a Syndrome? Answer from an Expression Profiling Study. *PLoS One*. 2016;11(2):e0149052. doi:10.1371/journal.pone.0149052

13. Simon RM, Dobbin K. Experimental design of DNA microarray experiments. *Biotechniques*. 2003;Suppl:16-21. http://www.ncbi.nlm.nih.gov/pubmed/12664680.

14. Kural-Seyahi E, Fresko I, Seyahi N, et al. The long-term mortality and morbidity of Behçet syndrome: a 2-decade outcome survey of 387 patients followed at a dedicated center. *Medicine (Baltimore)*. 2003;82(1):60-76. doi:10.1097/00005792-200301000-00006

15. Leccese P, Yazici Y, Olivieri I. Behcet’s syndrome in nonendemic regions. *Curr Opin Rheumatol*. 2017;29(1):12-16. doi:10.1097/BOR.0000000000000349

16. Direskeneli H, Saruhan-Direskeneli G. Disease Mechanisms. In: *Behçet Syndrome*. Cham: Springer International Publishing; 2020:209-222. doi:10.1007/978-3-030-24131-5_15

17. Ziegler SF, Ramsdell F, Alderson MR. The activation antigen CD69. *Stem Cells*. 1994;12(5):456-465. doi:10.1002/stem.5530120502

18. Yu L, Yang F, Zhang F, et al. CD69 enhances immunosuppressive function of regulatory T-cells and attenuates colitis by prompting IL-10 production. *Cell Death Dis*. 2018;9(9):905. doi:10.1038/s41419-018-0927-9

19. Sancho D, Gómez M, Viedma F, et al. CD69 downregulates autoimmune reactivity through active transforming growth factor-beta production in collagen-induced arthritis. *J Clin Invest*. 2003;112(6):872-882. doi:10.1172/JCI19112

20. Opipari AW, Boguski MS, Dixit VM. The A20 cDNA induced by tumor necrosis factor alpha encodes a novel type of zinc finger protein. *J Biol Chem*. 1990;265(25):14705-14708. http://www.ncbi.nlm.nih.gov/pubmed/2118515.

21. Zhou Q, Wang H, Schwartz DM, et al. Loss-of-function mutations in TNFAIP3 leading to A20 haploinsufficiency cause an early-onset autoinflammatory disease. *Nat Genet*. 2016;48(1):67-73. doi:10.1038/ng.3459
